# A multi-domain graph convolutional network-based prediction model for personalized motor imagery action

**DOI:** 10.3389/fnins.2025.1637018

**Published:** 2025-10-29

**Authors:** Jiahao Ge, Jie Wang, Xiao Zheng, Mengfan Li, Fuyong Wang, Guizhi Xu

**Affiliations:** ^1^State Key Laboratory of Intelligent Power Distribution Equipment and System, School of Health Sciences and Biomedical Engineering, Hebei University of Technology, Tianjin, China; ^2^Tianjin Hospital, Tianjin, China; ^3^China Electronics Technology Group Corporation Optoelectronics Research Institute, Beijing, China; ^4^School of Electrical Engineering, Hebei University of Technology, Tianjin, China; ^5^School of Artificial Intelligence, Nankai University, Tianjin, China

**Keywords:** brain-computer interface, graph convolutional network, feature fusion, brain network, correlation between cognitive tasks and MI, MI prediction

## Abstract

Motor imagery (MI)-based brain-computer interfaces (BCIs) offer a novel method to decode action imagination. Our previous study demonstrated that actions play a key role in causing individual differences. Cognitive EEG signals showed a positive correlation with MI, reflecting these differences and providing a foundation for predicting suitable MI actions for each individual. This study aimed to propose a multi-domain graph convolutional network (M-GCN) for predicting personalized MI action using cognitive data. The M-GCN extracts time, frequency, and spatial domain features from cognitive tasks to construct multi-domain brain networks using different EEG quantization methods according to the characteristics of the three domains. Subsequently, the M-GCN utilizes spectral GCN to learn the topology relationship between EEG channels by analyzing functional connection strength. Finally, for each action, the M-GCN can accurately map cognitive data to the corresponding MI action and output a personalized action for each subject. A subject-independent decoding paradigm with leave-one-subject-out cross-validation is adopted to validate the model on ten subjects. Compared to baseline and single-domain models, the M-GCN achieves the highest prediction accuracy of 73.60% (*p* = 7.1 × 10^−3^), improving by 15.87% (*p* = 2.0 × 10^−4^) and by 7.2% (*p* = 4.0 × 10^−4^), respectively. This study proves that the M-GCN can precisely predict personalized MI actions, reflecting the efficiency of the multi-domain feature fusion based on cognitive tasks and GCN and offering a novel method for personalized BCI.

## 1 Introduction

Brain-computer interfaces (BCIs) are advanced and innovative systems that enable direct communication between humans and external devices by utilizing data encoded in the brain activity (Shi et al., 2024a,b). BCIs based on a motor imagery (MI) paradigm (MI-BCIs) are widely used due to their active rehabilitation training nature and objective outcomes (Zhao et al., 2024).

However, there are significant individual differences among different people, which increase the difficulty of MI-BCIs training (Wang et al., 2004), reducing EEG decoding accuracy (ACC) and significantly reducing the efficiency of the MI-BCIs system (Li et al., 2024). Therefore, discovering an indicator that quantifies individual differences in MI is crucial. In our previous study, it was found that action is an important factor affecting individual differences (Deng et al., 2024). Action refers to a type of movement sequence involving the displacement of a limb in space, typically associated with purposeful behaviors in daily life, such as drinking water or writing. When participants are asked to imagine action, characteristic neural patterns are elicited in relevant brain areas. Event-related desynchronization (ERD) and event-related synchronization (ERS) are typical manifestations of MI. The ERD and ERS features could be translated into rehabilitation assistive commands or interventions for re-establishing the rehabilitation circuit between the individual and the external environment (Shi et al., 2024a,b). Certain actions are easier for participants to effectively imagine, thereby yielding better ERD/ERS and enhancing decoding performance. Moreover, the classification ACC distinguishing left- and right-hand MI is usually used as an evaluation metric. The suitable action tends to exhibit more pronounced discriminative ERD/ERS patterns between the left and right hemispheric motor regions, which may be associated with higher accuracy in left- and right-hand classification (Blankertz et al., 2008; Lotte et al., 2018). A higher classification ACC inherently indicates that the subject's MI of the corresponding action is more effective and easier for the decoding model to recognize (Wolpaw et al., 2002). Thus, each action can be divided into weak and strong types by individually evaluating their performance. However, the most suitable action may vary across participants; it is necessary to construct a machine learning model to predict a suitable action for each participant.

Moreover, the lengthy experimental cycle of MI makes it impractical to determine the personalized action solely based on extensive MI experiments. Consequently, efficiently predicting the personalized action for each subject remains a significant challenge. A correlation between cognitive EEG and MI has also been found in our previous study (Deng et al., 2024). Specifically, cognitive EEG and MI evoked by the same action show a positive correlation. More precisely, the amplitude of event-related potentials (ERP) and the intensity of ERD/ERS during MI are higher under a suitable action, while unsuitable actions reduce both. In contrast to MI, cognitive tasks offer a more effective approach by testing subjects' encoding and recollection of information over a short period, thereby enabling the rapid identification of the most suitable MI action without imposing an additional training burden. Based on the above, analyzing subjects' cognitive EEG to predict the most suitable MI action is an effective approach.

Considering that cognitive task and MI are both brain neural activities involving the collaboration of multiple brain regions, building a network model can concretely quantify multi-region EEG signals to achieve more precise prediction. A graph convolutional network (GCN) is a practical approach to address the problem mentioned above by applying the traditional discrete convolution concept to the graph structure. The GCN enables the extraction of spectral domain representations of different node feature information on the graph, fully capturing both the intrinsic features of the nodes and their topological relationships. EEG data are collected through multi-channel electrodes, where the EEG of each channel represents the neural activity of the specific brain region it covers. Thus, multi-channel EEG can be abstracted into a graph composed of point sets and edge sets. GCN, as a method suitable for analyzing multi-channel EEG, has been widely applied in EEG predicting and decoding that involve the collaboration of multiple brain regions. Du et al. (2022) proposed a frequency band attention graph convolutional adversarial neural network for emotion recognition, where channels within the frontal, temporal, and central lobes are represented as nodes in a graph, aiming to fully exploit the interactions between these regions. Wang et al. (2021b) introduced an attention-based multiscale convolutional neural network-dynamical GCN model to achieve driving fatigue detection. Leng et al. (2024) constructed a dense graph convolutional network to recognize stroke patients, with a focus on data from 12 channels in the frontal lobe for experimental analysis. Feng et al. (2021) proposed a GCN based on functional connectivity for motor intention decoding, and the brain function connection network was extracted from the 18 channels in the frontal, parietal, and occipital lobes. Wang et al. (2025) constructed a dynamic graph convolutional-capsule network that employs whole-brain region channels for MI decoding. These studies sufficiently demonstrate that the GCN is effective in EEG prediction and decoding based on multi-brain coordination.

Current studies primarily employ single-domain features or data as the input of GCN to decode the EEG, e.g., time domain, frequency domain, and spatial domain features. Sun et al. (2021) constructed an adaptive spatiotemporal GCN model based on time-domain dynamic features for MI classification. Pan et al. (2024) proposed a self-constructing graph neural network based on spatial features for emotion recognition and consciousness detection. Sun et al. (2022) introduced a complex network-based GCN model for major depressive disorder detection based on multi-band frequency domain features. However, the complexity and diversity of the EEG make it impossible for a single domain feature to capture the activity characteristics of the brain completely and accurately. The research shows that the fusion of different domains of features may better capture the intricate interactions between different EEG features and improve the model's ability to handle complex, multi-dimensional data. Wang fused three types of features—differential entropy, power spectral density, and functional brain network—to integrate the frequency domain and the spatial domain information of EEG signals for emotion recognition (Wang et al., 2023). Sun et al. (2022) extracted the PSD from six frequency bands and constructed multi-layer brain networks for each frequency band to train the model using the fused multi-frequency band brain networks for major depressive disorder detection. Xu et al. (2022) proposed a modified GCN for MI recognition by multiple features in the temporal-frequency-spatial domain to further improve the recognition performance. Chen et al. (2022) proposed a method that integrates frequency-domain features and brain connectivity features for cross-subject emotion recognition. Wang et al. (2020) employed filter-bank common spatial pattern and functional connectivity features to characterize the MI for a more comprehensive capture of the underlying neural mechanisms. Therefore, constructing GCN models based on the fusion of multi-domain features is a potential research direction.

Researchers usually adopt suitable brain synchronization quantification methods based on distribution characteristics of domain to construct brain network as input graph for GCN. Commonly used brain synchronization quantification methods include mutual information (Garn et al., 2015), phase locking value (PLV) (Yan et al., 2024), Manhattan distance (MD) (Ponten et al., 2007), Pearson correlation coefficient (PCC) (Yang and Li, 2025), and phase lag index (PLI) (Xu et al., 2022). Dasdemir analyzed functional brain connectivity of positive and negative emotions using the PLV based on frequency domain (Daşdemir, 2024). Shi utilized mutual information to measure the relationship between EEG channels and a GCN model, capturing the topological structure (Shi et al., 2024a,b). Wang established multiband functional connectivity matrices using the PLI (Wang et al., 2021a). Xue et al. (2024) used the Pearson correlation coefficient to analyze the brain connectivity of time-series signals from different channels. These examples fully illustrate the necessity of constructing multi-domain brain networks for distribution characteristics of different domains. Given this, integrating multi-domain brain networks may enhance the representational power of GCN models, leading to more precise personalized MI action prediction.

This study proposes an M-GCN model to predict personalized MI action. The M-GCN extracts time, frequency, and spatial domain features from cognitive tasks to construct multi-domain features, then constructs multi-domain brain networks by different EEG quantization methods according to the characteristics and distribution of different domains. Subsequently, the M-GCN learns features of brain region collaboration based on the topology connections of the brain network by spectral GCN. Finally, for each action, the M-GCN can accurately map the cognitive data to the corresponding MI action and output the personalized action for each subject. A subject-independent decoding paradigm with leave-one-subject-out cross-validation (LOSO-CV) is adopted to validate the model on ten subjects. The proposed framework is validated on ten participants and shows the highest prediction ACC (73.60%, *p* = 7.1 × 10^−3^). The above results prove that the M-GCN can achieve precise prediction of personalized MI action. Meanwhile, it also reflects that multi-domain feature fusion based on cognitive task and GCN are effective computational methods for personalized MI action prediction, providing a novel method for personalized BCI.

## 2 Materials and methods

**Figure 1:**
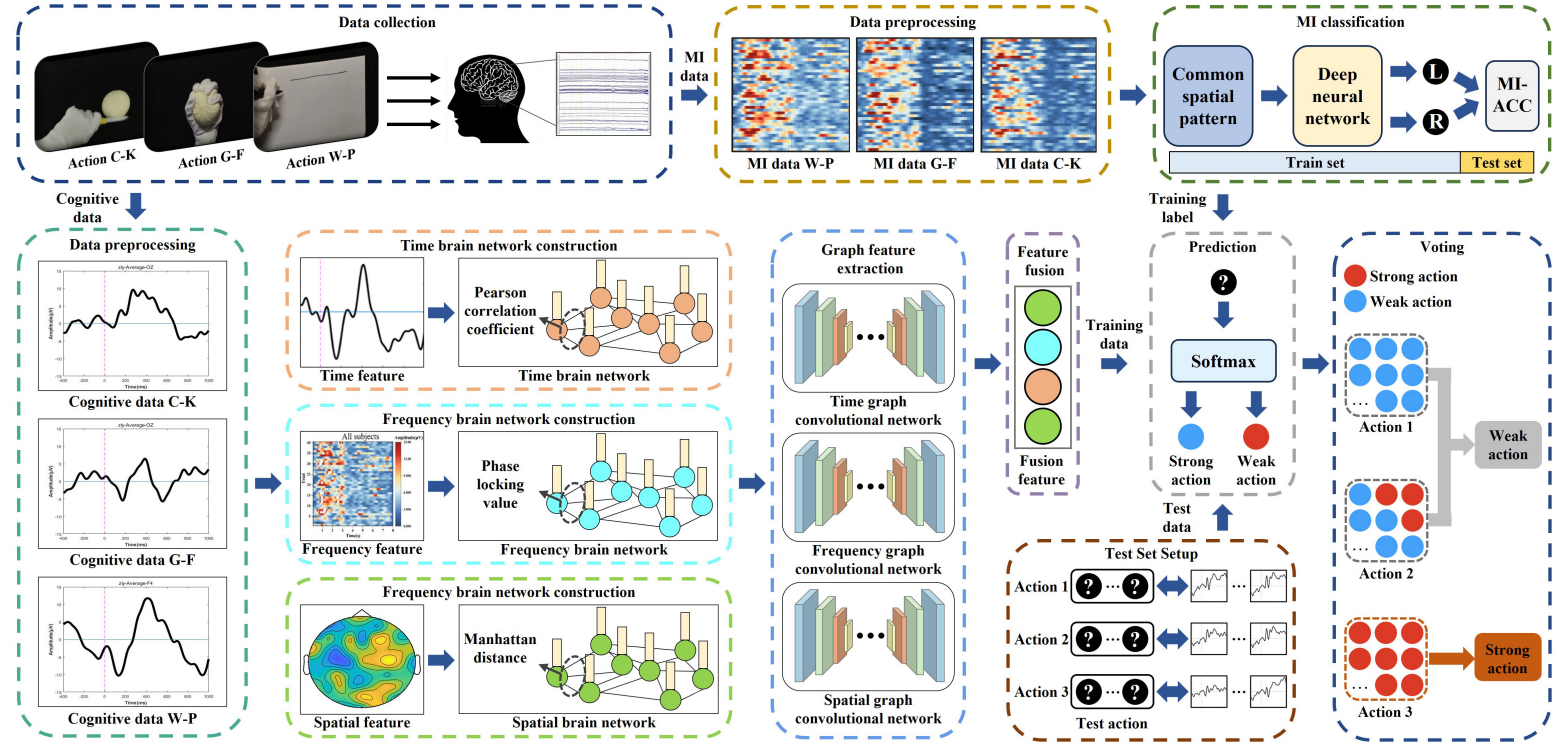
Framework of a multi-domain GCN model for predicting MI actions.

The framework design for M-GCN includes: (1) the construction of the multi-domain brain networks from cognitive data; (2) the building of the M-GCN to predict personalized MI action. The overall structure of the study is illustrated in Figure 1. Firstly, the cognitive data collected from previous studies are used to extract time, frequency, and spatial domain features to construct multi-domain features. Secondly, multi-domain brain networks are constructed by different EEG quantization methods according to the characteristics and distribution of different domains. Subsequently, spectral GCN is utilized to learn features of brain region collaboration based on the topological connections of the brain network. Finally, the M-GCN takes output features as samples and classifies labels according to the level of MI classification ACC to output the best performance action for individuals. Furthermore, the model is validated on ten participants using a subject-independent decoding paradigm with LOSO-CV.

### 2.1 Data collection

#### 2.1.1 Participants and acquisition device

A total of ten right-handed adults (three women, mean age = 24.3 ± 3.6 years) participated in this study (refers to S1–S10). The EEG acquisition device used a NeuroScan system with a sampling rate of 1,000 Hz. The 64 electrodes were arranged based on the 10–20 International System. The binaural mastoids were referenced, and channel AFz was grounded.

#### 2.1.2 Experimental paradigm

**Figure 2:**
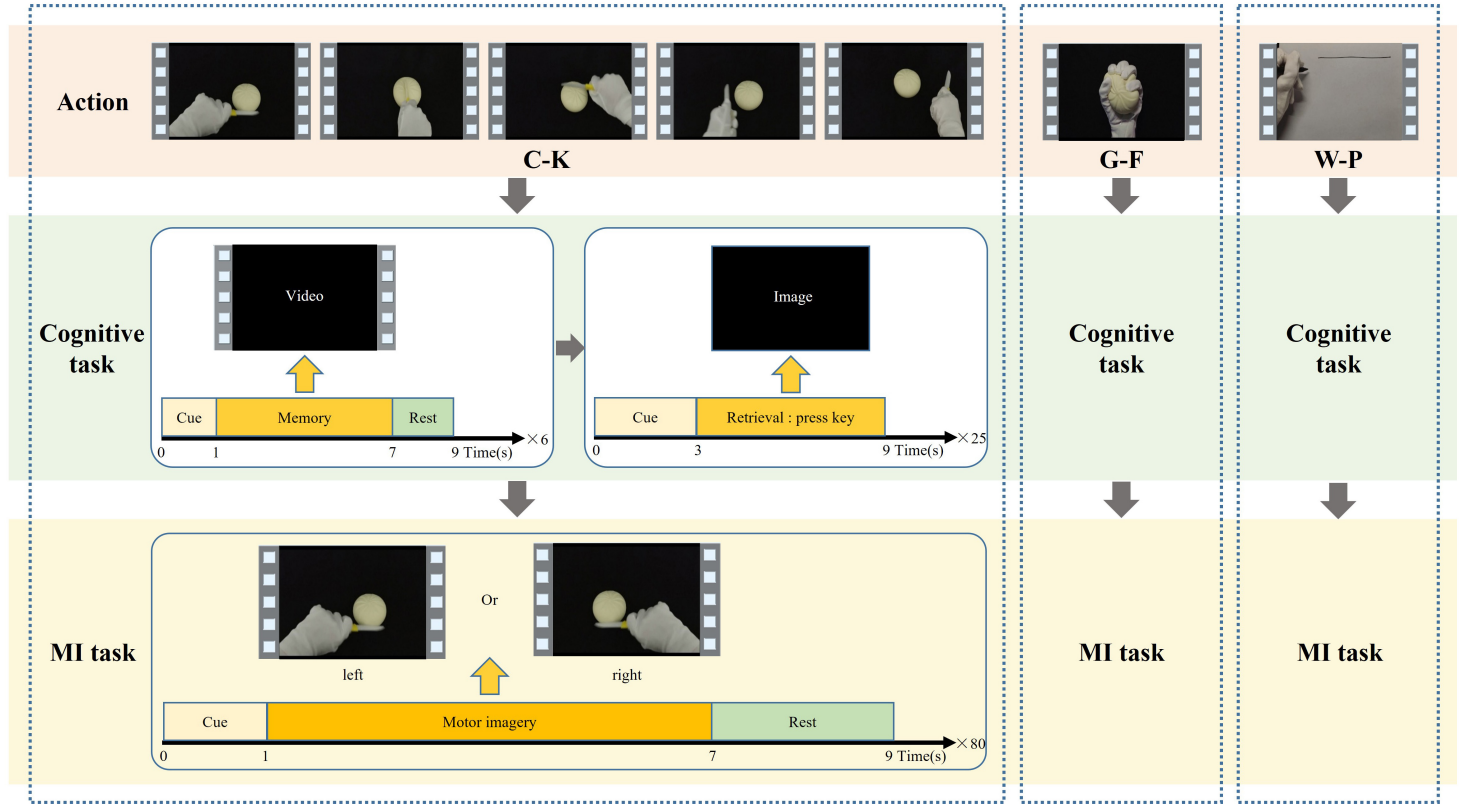
The schematic of the paradigm using C-K, G-F, and W-P daily action.

The cognitive data and MI data used in this study were both collected through the action observation-based delayed matching posture task (AO-DMPT) paradigm (Deng et al., 2024). The paradigm consists of a cognitive task and an MI task, as shown in Figure 2. By presenting action information in distinct forms, the two tasks enable the simultaneous collection of both cognitive and MI data from a subject. Three actions from daily life—cutting a ball with a knife (C-K), grasping a ball with fingers (G-F), and writing lines on paper with a pen (W-P)—are selected for the paradigm. Each action incorporates five postures for the left and right hands, respectively. And each subject completed both tasks for all three actions. For each action, both cognitive data and MI data are simultaneously obtained. The cognitive EEG data are employed to construct the input samples of the GCN. The MI data are exclusively utilized to determine the ground truth labels of each action, as MI data of each action represent the actual effect of imagery, ensuring that the model correctly maps cognitive data to the corresponding MI action.

##### 2.1.2.1 Cognitive task

Firstly, the cognitive task provides the subject with six action videos, including three left-hand postures and three right-hand postures, to remember the action. Each video stimulus is displayed once for 6 s, followed by a 3 s interval before the next video. Subsequently, 25 posture images extracted from the action videos are shown as visual stimuli. The subject is asked to match visual stimuli to memory by judging whether the posture has appeared in the initial video. Each image lasts for 6 s to allow the subject to make a judgment, and no image is repeated during the task. During this phase, the subject needs to be asked to encode action information and trigger memory to evoke cognitive EEG signals. For analysis, a time window from 0.2 s before to 1.0 s after the presentation of the action image is selected as cognitive data. Since every subject accomplishes three types of actions, the total trials for each subject are 75. The cognitive data is used to construct the brain network, which serves as the input to the M-GCN for the prediction of personalized MI action.

##### 2.1.2.2 MI task

The MI task repeatedly plays the action video to guide the subjects to MI. In the initial 0–2 s, a blank is displayed on the screen to allow the subject to rest. In 2–3 s, a white fixation cross appears at the center of the screen. In 3–9 s, an action video is played on the screen, accompanied by arrows indicating the corresponding directions of the left and right hands in the video. The combination of arrow and posture enables participants to perform MI from the correct direction. This phase consists of a total of 80 trials. During this phase, the subject needs to be guided to engage in autonomous imagery to generate MI signals, with the 6 s period after the presentation of each action video selected as MI data. Since every subject finishes three types of actions, the total number of MI data samples per subject is 240. The MI data are used for left- and right-hand classification to determine the mean MI-ACC of each action, which in turn defines the ground truth labels for each action.

This task involves identifying whether a given MI trial corresponds to left-hand or right-hand motor imagery. It is performed using a Common Spatial Pattern (CSP) and Deep Neural Network (DNN) model on the MI data. The mean MI-ACC for each action is used to define the ground truth label (strong or weak) for that action.

### 2.2 Data preprocessing

#### 2.2.1 Cognitive data preprocessing

Nineteen channels located near the supplementary motor area, motor area, and visual areas (FZ, FC3, FCZ, FC4, C5, C3, C1, CZ, C2, C4, C6, CP3, CP4, PO3, POZ, PO4, O1, OZ, O2) are selected to analyze cognitive data. This study uses a time window of 1.2 s to intercept the EEG data of 0.2 s before and 1 s after the image stimulus to analyze the cognitive data.

A third-order Butterworth bandpass filter from 0.5 to 20 Hz is used to effectively preserve cognitive potentials while removing high-frequency noise and ensuring accurate analysis of cognitive responses. The average data of the first 0.2 s is subtracted for baseline calibration. An independent component analysis algorithm was then applied to each channel and used to reduce electrooculogram and electromyography artifacts. The last 1 s period is defined as the raw cognitive data. A multichannel EEG signal *X*_*j*_ of a single trial is used as an example to describe the cognitive data after processing. Suppose that:


(1) $$\begin{array}{l} X_{j}=\;x_{h}^{t},\;h\;=\;1,\;2,\ldots ,19,\;t\;=\;1,\;2,\ldots ,\;1000 \end{array}$$

where *j* denotes the number of trials, *h* denotes the number of channels in the EEG signal, and *t* denotes the number of sample points in each channel. From each subject, 75 two-dimensional data vectors (19 × 1,000) can be obtained as input samples for the M-GCN.

#### 2.2.2 MI data preprocessing

Twelve channels located near the supplementary motion area (FC3, FCZ, FC4, C5, C3, C1, CZ, C2, C4, C6, CP3, and CP4) are selected to analyze MI data. The MI data is filtered using a fifth-order Butterworth bandpass filter in the range of 8 to 30 Hz. This range captures the sensorimotor rhythms, including both mu (8–13 Hz) and beta (13–30 Hz) bands, which are relevant for MI-based feature extraction. The classification of MI includes distinguishing between left- and right-hand actions.

The feature extraction by CSP that is a spatially filtered feature extraction method for classification tasks, aimed to extract the spatial components of each class from raw EEG data. The specific formula can be found in Qiu et al. (2017). Thus, a total of 80 feature vectors can be obtained from 80 MI trials for each subject in each action.

The DNN is used as a binary classifier to discriminate between the left and right hands. The DNN trains neuron weights repeatedly through forward propagation by setting multiple neurons in different layers and calibrates neuron weights through back propagation. The output of the DNN is expressed mathematically as follows:


(2) $$y=f(x,\theta )=f_{L-1}(W_{L-1}f_{L-2}\left(...f\left(W_{1}x+b\right)+b\right)$$

where *L* represents the number of network layers and is set to 4; θ represents the parameters, including the weights *w* and bias *b*. *w* and *b* are randomly initialized, and the learning rate is set to 0.05 and 0.01, respectively. Furthermore, the weight decay, sparsity regularization, and proportion of DNN are 0.05, 4, and 0.05, respectively. The structure of DNN is 4-100-50-10-2. DNN uses the feature vector after CSP extraction as input. The DNN output is the classification of the left and the right hand. In this study, a ten-fold cross-validation is adopted to yield an average ACC. The 80 trials are divided into 72 training trials and 8 test trials, with an equal number of left- and right-hand MI trials in each set. The MI-ACC for different actions are used to evaluate the ground truth label of each action, with higher ACC indicating a stronger action, while the remaining actions are considered weak.

Therefore, for each action of each subject, the corresponding sample constructed from cognitive data and its label determined from MI data can be obtained, ensuring that the GCN is trained on informative representations with reliable ground truth labels. Each subject completes a total of 75 cognitive task trials. These trials are classified into 25 strong action trials and 50 weak action trials according to the MI classification ACC for the left and right hands. The cognitive data from these trials are used as input samples for the model, while the corresponding labels derived from the MI data serve as the labels, enabling the model to learn the mapping from cognitive data to the actual MI action.

### 2.3 Multi-domain brain network construction

Brain networks constructed from cognitive data consist of plenty of nodes and edges; 19 channels located near the supplementary motor area, motor area, and visual areas are set as nodes. Various EEG quantization methods according to the characteristics of different domains are selected to determine the edges. Cognitive data obtained during the cognitive task are used to construct three types of brain networks: time domain, frequency domain, and spatial domain networks.

#### 2.3.1 Time domain brain network construction

Down-sampling is a widely used data dimensionality reduction method, typically employed to reduce the sampling rate or data size of a signal while preserving its key features. Thus, down-sampling is applied only for the time domain feature extraction to obtain informative representations. Multiple down-sampling factors are tested to evaluate their impact on model performance. A factor of 40 is found to provide a balance between data reduction and retention of relevant EEG information, enabling efficient training of deep learning-based models without significant loss of ACC. Thus, the data are averaged and down-sampled with a down-sampling rate of 40, meaning that the retained data points are averages over the nearest 40 data points. For each of the 19 channels, 40 points could be derived, and a two-dimensional feature matrix (19 × 40) can be obtained for each trial as a time domain feature.

For the time domain feature, the edges can be determined via PCC. The PCC, a statistical method widely used to measure the linear correlation between two variables, can be used to characterize the EEG synchronization between two time series, the time domain feature *D*_*j*_ of a single trial is used as an example to show the specific equation:


(3) $$w_{h1,h2}\;=\;|\frac{\sum_{t=1}^{T}\left(d_{h1}^{t}-{\bar{d}}_{h1})(d_{h2}^{t}-{\bar{d}}_{h2}\right)}{\sqrt{\sum_{t=1}^{T}{\left(d_{h1}^{t}-{\bar{d}}_{h1}\right)}^{2}}\sqrt{\sum_{t=1}^{T}{\left(x_{h2}^{t}-{\bar{d}}_{h2}\right)}^{2}}}|$$

Where $d_{h1}^{t}$, $d_{h2}^{t}$ are the time features vectors from the channel *h*1 and *h*2; *T* is the total number of time features on a channel, **w**_**h1, h2**_ ε [0, 1] can quantify the relationship between two channels and assess the strength of their correlation; an adjacency matrix (19 × 19) can be obtained for each trial.

By combining the node feature matrix and the adjacency matrix, the time domain brain network could be defined. Two components of the brain network, the adjacency matrix *A* and the node feature matrix *x*, together constitute the input for each trial to the M-GCN model.

#### 2.3.2 Frequency domain brain network construction

The wavelet transform (DWT) is a Fourier transform-based time-frequency analysis tool. The wavelet coefficients can represent signal information in both the time and frequency domains. The DWT can be expressed using the following Equation:


(4) $$\begin{array}{l} W\left(j,k\right)=\sum_{N=0}^{M-1}x_{h}^{n}•\psi_{j,k}^{*}\left(n\right) \end{array}$$

Where $x_{h}^{n}$ is a signal (sequence) of length *n*; $\psi_{j,k}^{*}\left(n\right)$ is scaling the wavelet function. Four levels of Daubechies wavelets are used to decompose EEG into four band: 1–4 Hz, 4–8 Hz, 8–13 Hz and 13–20 Hz. Furthermore, for the four frequency bands, the PSD features are captured via the Welch method, a two-dimensional feature matrix (19 × 4) can be obtained for each trial as frequency feature.

The PLV is an effective index used to quantify the phase synchronization between two group of frequency features, which is a typical method for analyzing brain functional connections. The PLV of frequency features is defined as follows:


(5) $$\begin{array}{l} PLV=\frac{1}{T}|\sum_{t=1}^{T}e^{i\phi \left(t\right)}| \end{array}$$

Where ϕ represents the phase difference, calculated between channel h1 and h2, and an adjacency matrix (19 × 19) can be obtained for each trial. Thus, by combining the node feature matrix and the adjacency matrix, the frequency domain brain network is defined.

#### 2.3.3 Spatial domain brain network construction

CSP, a widely used spatial filtering feature extraction algorithm, is used to extract spatial distribution components. Its basic principle involves diagonalizing matrices to identify a set of optimal spatial filters for projection, which maximizes the variance difference between the two types of signals, thereby obtaining feature vectors with high discrimination.

Given the number of EEG channels *h*, the number of samples per channel *t*, the *h* by *t* matrix of EEG data of the strong action, and the *h* by *t* matrix of EEG data of the weak action, the CSP computes the n by n matrix *W* such that the normalized variances: *var*(*W*_*i*_*Strong*) = 1- *var*(*W*_*i*_*Weak*). Where *W*_*i*_ represents the *i*^*th*^ row of *W*. By taking the rows of *W* for which those variances are the closest to 0/1, one obtains the directions of optimal discriminability through variance. Finally, the most discriminative and non-redundant information could be extracted from the EEG signal; a two-dimensional feature matrix (19 × 1) can be obtained for each trial as a spatial feature.

The MD is a distance measurement method in mathematics and computing. MD can provide a simple and computationally cheap distance measurement method, which is suitable for dealing with high-dimensional features to evaluate the degree of information sharing between different CSP features. MD can be computed using the equation:


(6) $$L_{h1,h2}=\left|s_{h1}-s_{h2}\right|$$

Where *s*_*h*1_, *s*_*h*2_ are the spatial features vectors from the channel *h*1 and *h*2. Considering the large values of the MD, normalization is necessary. To achieve this, we employ min-max normalization, thereby mapping MD values into the [0, 1] range. The specific equation is defined as follows:


(7) $$\begin{array}{l} X_{normalized}=\frac{x-min\left(x\right)}{\max\left(x\right)-min\left(x\right)} \end{array}$$

An adjacency matrix (19 × 19) can be obtained for each trial. Thus, by combining the node feature matrix and the adjacency matrix, the spatial domain brain network is defined.

Finally, for each trial of each subject, time-domain, frequency-domain, and spatial-domain brain networks are obtained, respectively, as inputs to the model.

### 2.4 Multi-domain graph convolutional network prediction model

**Figure 3:**
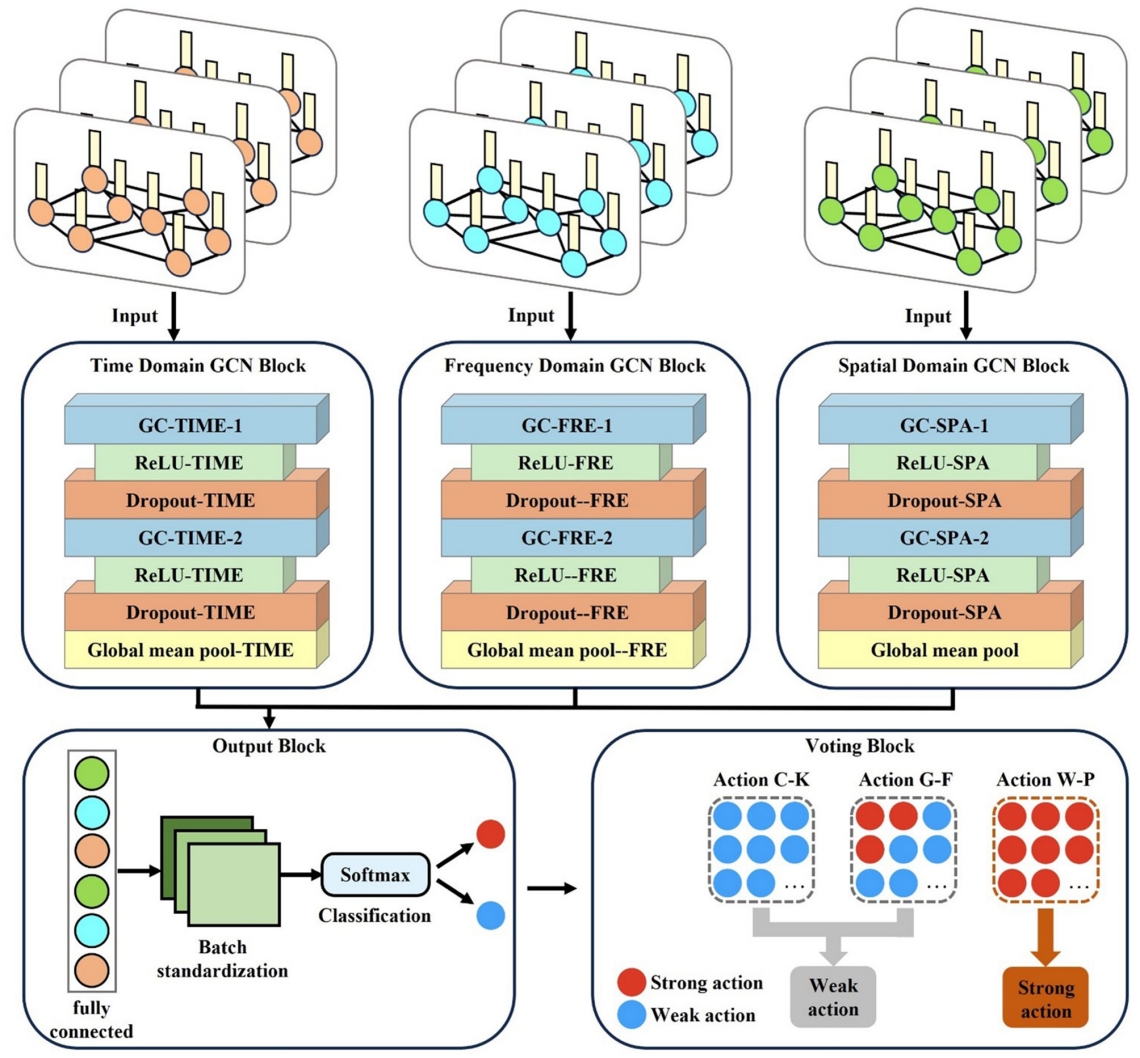
Structure of a multi-domain graph convolutional network prediction model.

As shown in Figure 3, the M-GCN comprises five blocks: the time domain GCN block (TDN), the frequency domain GCN block (FDN), and the spatial (SDN) domain GCN block process brain networks from the corresponding domain separately, learning the functional connections between EEG channels via spectral graph convolution (GC) layers, rectified linear unit (ReLU), and dropout. The output block integrates multi-domain features through a fully connected layer, normalizes them via batch standardization, and classifies using SoftMax. The voting bloc determines the final action based on majority votes. Additional model details are provided in Table 1.

**Table 1:** Graph convolution network based on multi-domain brain networks.

**Module**	**Type**	**Parameters (activation function)**	**Output shape**
TDN	Input		19.40
	GC-TIME-1	32(ReLU)	19.32
	Dropout-TIME	0.5	19.32
	GC- TIME−2	16(ReLU)	19.16
	Dropout-TIME	0.5	19.16
	Global mean pool-TIME	Mean value of each node	16
FDN	Input		19.4
	GC-FRE-1	32(ReLU)	19.32
	Dropout-FRE	0.5	19.32
	GC-FRE−2	16(ReLU)	19.16
	Dropout-FRE	0.5	19.16
	Global mean pool-FRE	Mean value of each node	16
SDN	Input		19.1
	GC-SPA-1	32(ReLU)	19.32
	Dropout-SPA	0.5	19.32
	GC-SPA-2	16(ReLU)	19.16
	Dropout-SPA	0.5	19.16
	Global mean pool-SPA	Mean value of each node	16
Output block	Concatenate	–	16^*^3
	Batch standardization		
	SoftMax	2	2

The asterisk (*) indicates feature concatenation across the three domain branches (time, frequency, and spatial), resulting in a combined feature dimension.

As depicted in Figure 3, for each input multi-domain brain network, the time domain, frequency domain, and spatial domain brain networks are input into the TDN, FDN, and SDN, respectively. Each network is first processed through a GC layer based on spectral GC.

Assume that each input brain network is represented as a graph *G* = {*V, E, A, x*}, where *V* represents the set of nodes with *h* feature vectors, and *E* stands for the set of edges. *A* ε R^*S*×*S*^ is a weighted adjacency matrix that represents the connection strength between two nodes, where *S* denotes the number of nodes in the graph, corresponding to the number of EEG channels in the brain network. A node feature matrix *x* ε R^*S*×*f*^ is defined for each trial, where *f* denotes the feature dimension of each node. The graph Laplacian of *G* is defined in the following way:


(8) $$\begin{array}{l} L=D-A \end{array}$$

Where *D* ε R^*S*×*S*^ is the degree matrix of *G*; each matrix entries in *D* can be calculated by the equation:


(9) $$\begin{array}{l} D_{ii}=\sum_{j}A_{ij} \end{array}$$

where *i* and *j* denote the indices of nodes in the graph, each element in the diagonal matrix *D* indicates the number of edges associated with this node and the degree of performance of this node, i.e., the degree of the node. The normalized equivalent of *L* is given as follows:


(10) $$\begin{array}{l} \tilde{L}=I-D^{-\frac{1}{2}}AD^{-\frac{1}{2}} \end{array}$$

Where *I* ε R^*S*×*S*^ is an identity matrix. The real symmetric matrix $\tilde{L}$ can be diagonalized as:


(11) $$\begin{array}{l} \tilde{L}=U\wedge U^{T} \end{array}$$

Where *U* ε R^*S*×*S*^ represents a matrix of eigenvectors; Λ ε R^*S*×*S*^ is a diagonal matrix whose elements are composed of eigenvalues λ_1_, λ_2_,...,λ_*S*_, respectively.

Assuming that a graph signal is *x*, the spectral graph convolutional operation can be formulated as follows:


(12) $$\begin{array}{l} g_{\theta }^{*}x=Ug_{\theta }\left(\wedge \right)Ux^{T}x \end{array}$$

Where *g*_θ_ represents a graph filter constrained by θ, *g*_θ_(∧) can be calculated by the equation:


(13) $$g_{\theta }\left(\wedge \right)=\{\begin{array}{c} g\left({\text{λ}}_{1}\right)\;\cdots \;0\\ \;\vdots \;\ddots \;\vdots \\ \;0\;\cdots \;g({\text{λ}}_{S}) \end{array}$$

Since directly decomposing the eigenvalues of L is very time-consuming, *K*-order Chebyshev polynomials can be utilized to approximate *g*_θ_, which is calculated by the following equation:


(14) $$\begin{array}{l} g_{\theta }^{*}x\approx \sum_{m=0}^{K}\theta_{m}T_{m}\left(\tilde{\wedge }\right)x \end{array}$$

Where $T_{m}\left(\tilde{\wedge }\right)$ is the *m*-order of the Chebyshev polynomial, and the formula of $\tilde{\wedge }$ is as follows:


(15) $$\begin{array}{l} \tilde{\wedge }=\frac{2\wedge }{\lambda_{\max}}-I \end{array}$$

Where λ_max_ is the maximum eigenvalue of $\tilde{L}$. This calculation ensures that the eigenvalue lies within [−1, 1], which is required for the stable computation of Chebyshev polynomials. For the sake of efficiency and computational ACC, the Chebyshev polynomial order is set to 1 in spectral convolution. The propagation rule of each convolutional layer in GCN is given as follows:


(16) $$\begin{array}{l} G^{\left(l+1\right)}=\sigma \left({\tilde{D}}^{-\frac{1}{2}}Ã{\tilde{D}}^{-\frac{1}{2}}G^{\left(l\right)}W^{\left(l\right)}\right) \end{array}$$

Where *G*^(*l*)^ and *G*^(*l*+1)^ denote the input and output of the (*l*)th graph convolution (GC) layer, respectively; *W*^(*l*)^ represents the parameter matrix of each layer; σ is the activation function. In this study, the ReLU function is adopted as σ, defined as:


(17) $$\begin{array}{l} f\left(x\right)=max\left(0,x\right) \end{array}$$

The primary goal of ReLU is to introduce nonlinearity so that neural networks can learn complex nonlinear features. To capture hierarchical features across dimensions while preventing overfitting, two GC layers with 32 and 16 hidden units, respectively, are configured for each domain branch (TDN/FDN/SDN) to progressively extract higher-level representations from the input graphs. Each graph convolutional layer is followed by a dropout layer to prevent overfitting and enhance model generalization. A dropout rate of 0.5 is applied after each GC layer to reduce overfitting. By randomly cropping a portion of neurons during training, it effectively alleviates overfitting of features. Subsequently, a mean pooling operation is applied over all nodes to aggregate their features and generate advanced features.

All learned features from the three branches are fed into the output block, which is composed of a fully connected layer, a batch standardization layer, and a SoftMax layer. The fully connected layer integrates the features extracted by the TDN, FDN, and SDN; the batch standardization layer normalizes the data based on the mean and standard deviation to eliminate amplitude discrepancies in the advanced features; and then the SoftMax layer outputs the final binary prediction of whether the input cognitive data of each trial corresponds to a “strong” or “weak” MI action for the subject. This result is then passed to the voting bloc. During M-GCN training, the learning rates and number of training epochs are set to 0.001 and 1000, respectively. Empirical observations confirmed stable training dynamics, with loss convergence typically achieved by epoch 800.

Finally, the voting module conducts a vote on all the discrimination results of a subject for the three actions. Specifically, the module sequentially processes the cognitive data corresponding to each action through the model, and this procedure is repeated independently for each of the three actions. Then, the module counts the number of “strong” predictions for each action and compares these counts across the three actions. The action with the highest number of “strong” predictions is selected as the subject's personalized MI action, assuming it achieves the best MI effect.

To evaluate the ability of the proposed model across different individuals, the validation is performed on ten participants. A subject-independent decoding paradigm by applying a LOSO-CV is adopted to evaluate the generalization ability of the proposed model. In the training phase, cognitive EEG data from all but one subject are used as the model inputs, and the corresponding MI data of these subjects serve to provide the ground truth labels. In the testing phase, only the cognitive EEG data of the remaining left-out subject are fed into the model, while their ground truth labels from MI data are used solely for performance evaluation. The LOSO-CV is repeated until every subject has been used once as the test set.

### 2.5 Evaluation metrics

#### 2.5.1 Accuracy metrics

For binary classification, data can be classified into four categories based on true and predicted values: true-positives (TP), false-positives (FP), true-negatives (TN), and false-negatives (FN), where positive denotes that the predicted action is consistent with the real action and negative denotes the opposite. Table 2 shows the evaluation matrix.

**Table 2:** Evaluation matrix.

		**Predicted label**	
		**Strong action**	**Weak action**
True label	Strong action	TP	FN
	Weak action	FP	TN

F1, sensitivity, specificity, and ACC are also commonly applied to evaluate the performance of the model. The equations for them are as follows:


(18) $$\begin{array}{l} F1=\frac{2TP}{2TP+FP+FN} \end{array}$$

(19) $$\begin{array}{l} Sensitivity=\frac{TP}{TP+FN} \end{array}$$

(20) $$\begin{array}{l} Specificity=\frac{TN}{FP+TN} \end{array}$$

(21) $$\begin{array}{l} Accuracy=\frac{TP+TN}{TP+FP+TN+FN} \end{array}$$

#### 2.5.2 Kappa coefficient

The Kappa coefficient is an index used to evaluate the consistency and effect of a classifier. Consistency is whether the predicted results of the model are consistent with the actual prediction results (Difonzo et al., 2022). It is calculated as follows:


(22) $$\begin{array}{l} Kappa=\frac{Accuracy-p_{e}}{1-p_{e}} \end{array}$$

(23) $$\begin{array}{l} p_{e}=\frac{\left(TP+TN\right)\left(TP+FP\right)+\left(FP+TN\right)\left(FN+TN\right)}{{\left(TP+FP+TN+FN\right)}^{2}} \end{array}$$

#### 2.5.3 Clustering coefficient

For brain networks, the clustering coefficient is a key metric used to measure the local connectivity and modular structure of a network. Furthermore, the average of the clustering coefficients of all n describes the density of all clusters in the entire network. Its value ranges between 0 and 1. The larger the C, the more the nodes in the network are clustered together, indicating a higher local clustering within the network. The specific equation is defined as follows:


(24) $$\begin{array}{l} C=\frac{1}{h}\sum_{i=1}^{h}C_{i}=\frac{1}{h}\sum_{i=1}^{h}\frac{E_{i}}{k_{i}\left(k_{i}-1\right)/2} \end{array}$$

Where *C*_*i*_ represents the clustering coefficient of channel *I*; *E*_*i*_ represents the number of neighboring nodes directly connected to channel *i*; *k*_*i*_ is the degree of channel *i*.

### 2.6 Control models

To ensure a more comprehensive and fair comparison, this study compares M-GCN with five baseline models. More details are as follows:

***Logistic regression (LR)***: A traditional linear model that is often used as a baseline for classification tasks due to its interpretability and effectiveness in modeling linear relationships (Deng et al., 2024).
***EEGNet***: A compact and efficient convolutional neural network designed for EEG decoding, capable of extracting spatial and temporal features from EEG signals effectively (Cecotti and Graser, 2011).
***Convolutional neural network (CNN)***: It can automatically learn hierarchical features from raw data through convolutional layers (Lawhern et al., 2018).
***GCN-LSTM***: A graph-based hybrid deep learning model that combines GCN with long short-term memory (LSTM) networks, enabling it to capture both the complex spatial dependencies among EEG channels and the temporal dynamics of neural signals (Gosala et al., 2025).
***SAST-GCN***: A segmentation adaptive spatial-temporal GCN based on the spatial-temporal structure of multi-channel EEG signals. It can effectively improve the recognition performance by reflecting the dynamic connection relationship between EEG channels more accurately (Lu et al., 2022).

## 3 Result

### 3.1 Accuracy analysis of distinguishing strong and weak action

#### 3.1.1 Accuracy with different models

The ACC, a crucial criterion in the field of BCIs, representing the ratio of correctly detected trials to the total number of trials, is utilized to verify the prediction performance of models. The significance test method uses a *P*-value to represent statistical significance.

**Figure 4:**
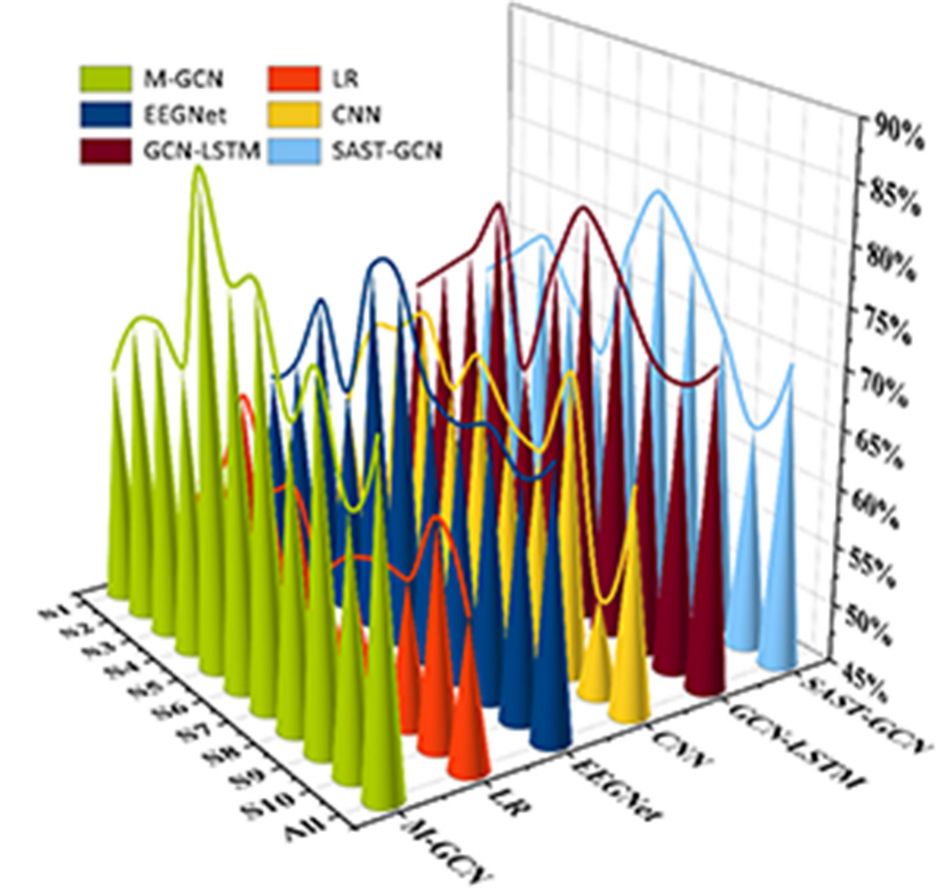
The prediction ACC for different models.

This study implements personalized MI prediction for ten subjects using different models separately. As shown in Figure 4, the M-GCN outperforms five baselines, i.e., LR, EEGNet, CNN, GCN-LSTM, and SAST-GCN. When trained by multi-domain features, the M-GCN reaches the highest average prediction ACC of 73.60% (*p* = 7.1 × 10^−3^), improving by 15.87% (*p* = 2.0 × 10^−4^) compared to LR, by 9.47% (*p* = 1.3 × 10^−3^) compared to CNN, by 4.27% (*p* = 1.0 × 10^−3^) compared to the EEGNet, by 1.60% (*p* = 2.32 × 10^−2^) compared to the GCN-LSTM, and by 3.06% (*p* = 4.65 × 10^−2^) compared to the SAST-GCN. This means that GCN can better capture the intricate interactions between different EEG features from multi-domain features and effectively improve the performance of personalized MI action prediction.

#### 3.1.2 Accuracy with different domains

To validate whether the fusion of the multi-domain influences the performance of personalized MI action prediction, this study sets up four groups with varying domain features. Table 3 lists their corresponding F1, sensitivity, specificity, and ACC. The performance difference is also observed from our results. All models show improved metrics and achieve the state-of-the-art performance in the multi-domain setting. Comparing different single-domain features in GCN, the multi-domain exhibits the highest ACC of 73.60% (*p* = 7.1 × 10^−3^). Meanwhile, multi-domain features improve by 7.20% (*p* = 4.0 × 10^−4^), 6.8% (*p* = 4.0 × 10^−3^), and 5.73% (6.00 × 10^−4^) compared to the time, frequency, and spatial domains, respectively. It can also be seen that the multi-domain feature performs well in terms of F1 score, sensitivity, and specificity compared with the other single-domain features. The results demonstrate that the fusion of different domains of features may better capture the intricate interactions between different EEG features and improve the performance of personalized MI action prediction. In general, the prediction ACC decreases as the number of domains in a multi-domain feature decreases. While the advantage of the GCN is better capturing the intricate interactions between different EEG features from multi-domain features, when only single-domains are used like frequency domain feature, the GCN still retains the highest ACC of 66.80%, improving by 11.73% (*p* = 2.0 × 10^−3^) compared to LR, by 3.47% (*p* = 4.36 × 10^−2^) compared to EEGNet, by 5.20% (*p* = 1.52 × 10^−2^) compared to the CNN, by 0.79% (*p* = 3.28 × 10^−2^) compared to the GCN-LSTM and by 2.80% (*p* = 3.2 × 10^−3^) compared to the SAST-GCN. This indicates that even when limited to a single domain, the GCN maintains a strong predictive capability by effectively leveraging the intrinsic structure of EEG features. Therefore, the M-GCN is demonstrated to be a generalization model that can use single-domain features to achieve personalized MI action prediction.

**Table 3:** Performance metrics for different domains.

**Model**	**Domain**	**F1**	**Sensitivity**	**Specificity**	**ACC**
M-GCN	Multi-domain	*0.66*	*0.76*	*0.72*	*73.60%*
GCN	Time domain	0.59	0.72	0.64	66.40%
	Frequency domain	0.55	0.64	0.66	66.80%
	Spatial domain	0.61	0.76	0.64	67.87%
LR	Multi-domain	0.47	0.56	0.58	57.73%
	Time domain	0.43	0.52	0.54	53.47%
	Frequency domain	0.43	0.52	0.56	55.07%
	Spatial domain	0.46	0.56	0.56	56.40%
EEGNet	Multi-domain	0.60	0.73	0.65	68.00%
	Time domain	0.53	0.68	0.56	61.73%
	Frequency domain	0.57	0.72	0.59	63.33%
	Spatial domain	0.52	0.64	0.60	66.53%
CNN	Multi-domain	0.56	0.68	0.62	64.13%
	Time domain	0.49	0.60	0.58	58.93%
	Frequency domain	0.52	0.64	0.60	61.60%
	Spatial domain	0.52	0.64	0.58	63.20%
GCN-LSTM	Multi-domain	0.64	0.76	0.70	72.00%
	Time domain	0.56	0.68	0.62	64.00%
	Frequency domain	0.53	0.64	0.62	62.67%
	Spatial domain	0.58	0.72	0.62	65.33%
SAST-GCN	Multi-domain	0.63	0.74	0.69	70.67%
	Time domain	0.53	0.64	0.62	62.67%
	Frequency domain	0.54	0.64	0.64	64.00%
	Spatial domain	0.57	0.72	0.60	64.00%

Italic values indicate the best performance achieved under the corresponding domain.

### 3.2 Confusion matrices and kappa coefficient

**Figure 5:**
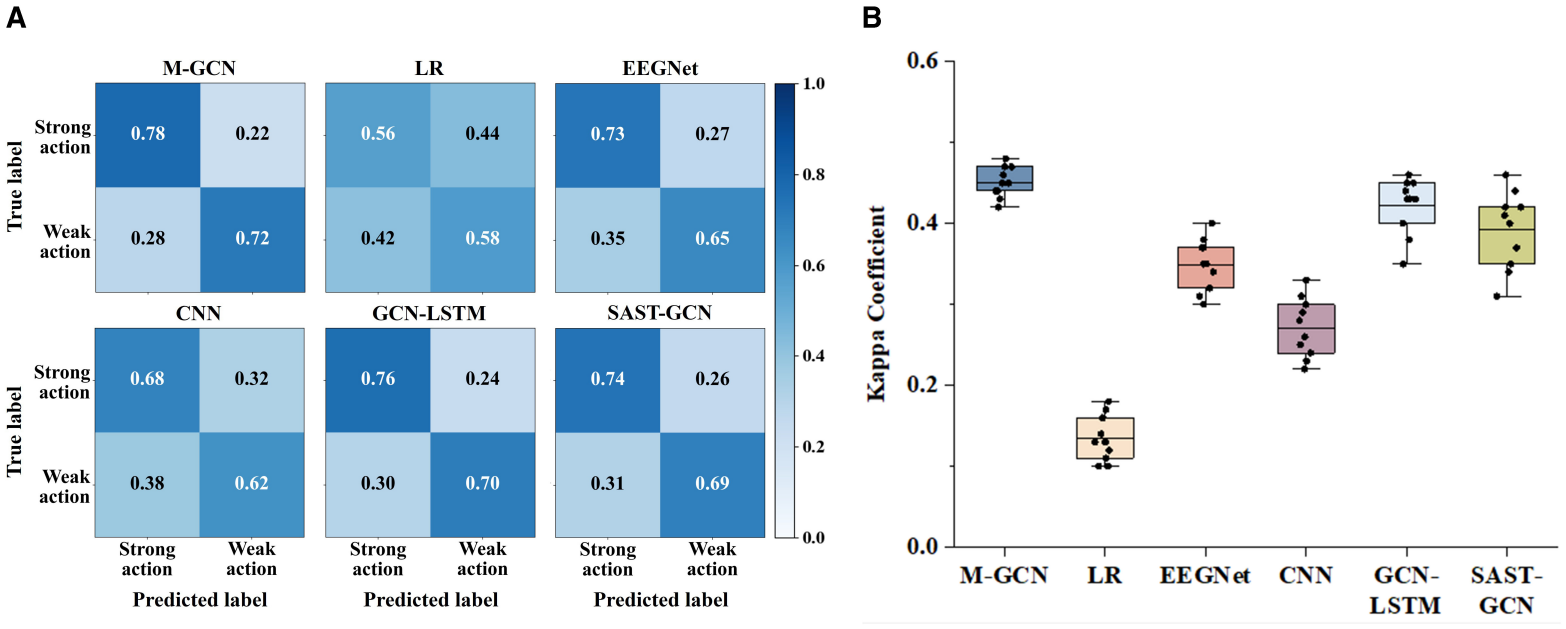
**(A)** Confusion matrices for the multi-domain features of M-GCN and the baseline model. **(B)** Kappa coefficient for the multi-domain features of M-GCN and the baseline model.

Confusion matrices and the kappa coefficient are utilized to verify the predicted consistency of the model. Figure 5A shows the confusion matrices of the six models for multi-domain features, arranged from top to bottom. The confusion matrices include the predictions for the categories of strong action and weak action, and the number of correct predictions is indicated on the diagonal. Compared with the LR, EEGNet, CNN, GCN-LSTM, and SAST-GCN. M-GCN shows higher TP and TN. This indicates that M-GCN in this study outperforms the other three methods in terms of prediction performance. Meanwhile, Figure 5B lists the kappa coefficients with four models, demonstrating their superior consistency and reliability in prediction. The significant improvement in kappa indicates that incorporating multi-domain features into GCN-based modeling effectively captures the intricate relationships within EEG data, resulting in improved predictive performance.

### 3.3 Consistency between predicted and ground truth MI actions

Action exhibits a consistent pattern of neural activity across both cognitive and MI EEG signals. Although the specific strong and weak actions may differ across participants, the model is still able to achieve the precise prediction of suitable MI action.

**Figure 6:**
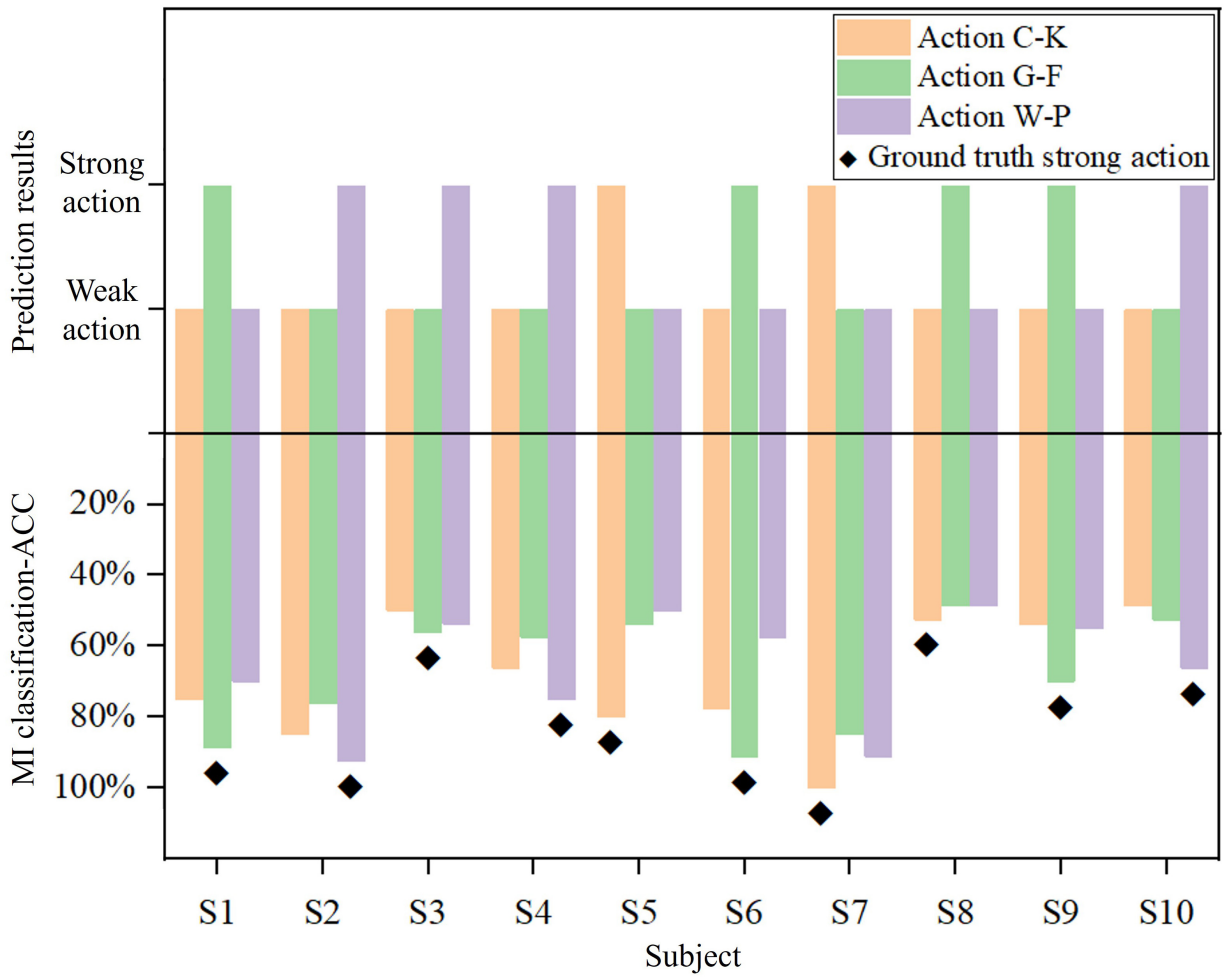
Comparison of true MI classification-ACC and prediction results across different actions for each subject.

As shown in Figure 6, the prediction results and the true MI classification ACC of three actions are compared for each participant, where the upper panel presents the prediction of the M-GCN, and the lower panel illustrates the classification ACC of left- vs. right-hand MI under different action conditions. First, for most participants, the predicted strong action is consistent with the action that achieves the highest MI-ACC, indicating that the proposed model can effectively capture the individual differences and provide reliable personalized action selection. Furthermore, there are individual differences in strong and weak actions, and each subject exhibits a distinct pattern. Finally, although subjects 3 and 8 exhibit inconsistencies in the results, this can be attributed to the small differences in MI-ACC among the three actions, so that the M-GCN could not clearly distinguish the optimal action, resulting in minor inconsistencies between the predicted and true strong actions. Overall, these findings demonstrate that the proposed M-GCN can effectively predict the most suitable MI action for each subject, corresponding well with the action with high ACC.

### 3.4 Multi-domain brain networks of cognitive data

**Figure 7:**
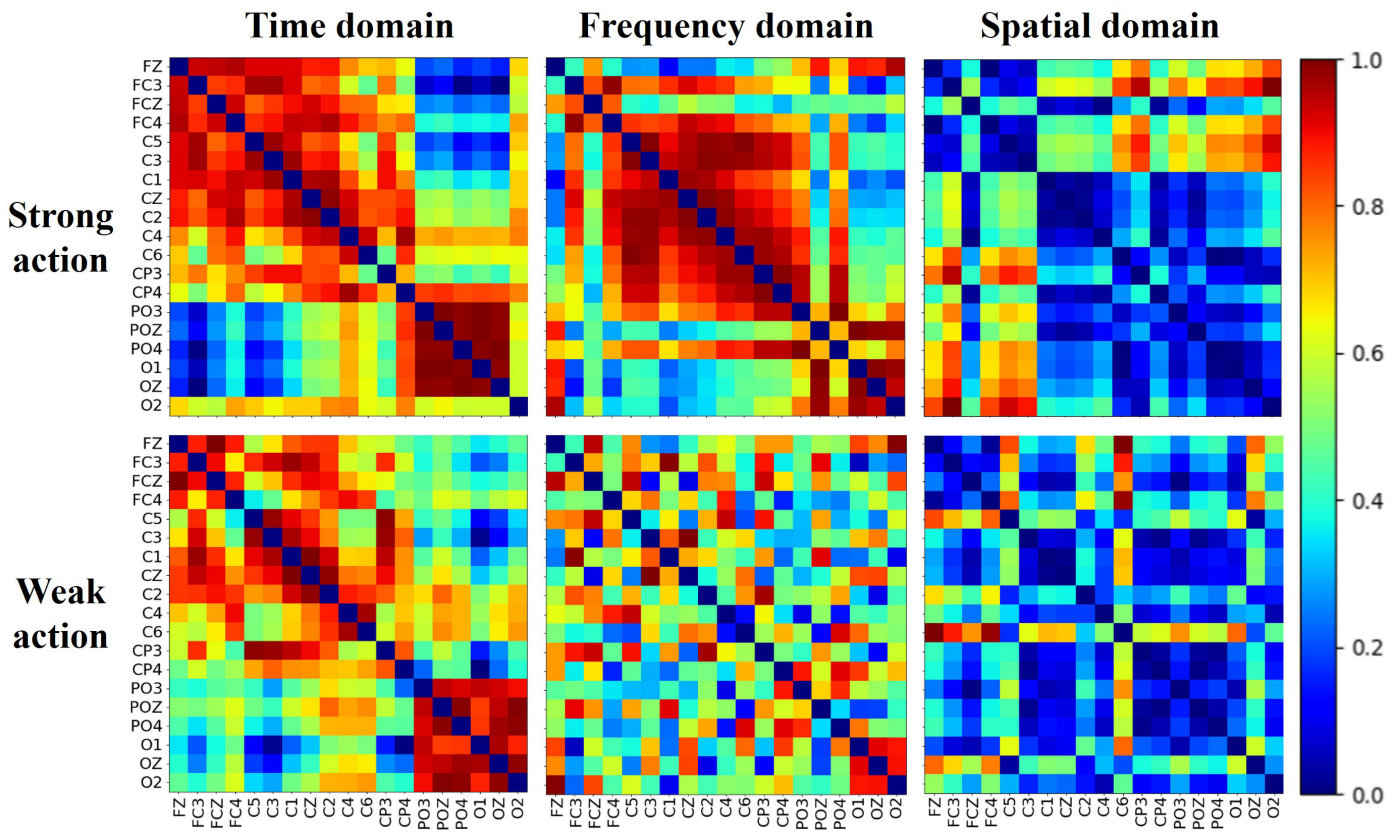
Multi-domain brain networks of strong and weak action obtained for all subjects.

The brain shows different network patterns for information processing in various situations. The brain network for different actions may reflect the characteristics across distinct brain regions. Figure 7 shows the normalized multi-domain brain network of the cognitive data of strong and weak actions, derived from the average of all subjects. Each subfigure in one subject represents one domain, and the color denotes the connection strength value between pairs of channels, with warm colors indicating stronger connections and cool colors indicating weaker connections. As shown in the figure, the networks perform various connectivity patterns in the different domains differ noticeably in terms of density, distribution, and regional interaction, which suggests that the brain network shows different behavioral characteristics in the different domains.

Moreover, within the same domain, the brain networks exhibit distinct topological structures between strong and weak action. In the time domain, strong actions exhibit denser and more uniformly high connectivity than weak actions, suggesting more synchronized temporal activity. In the frequency domain, strong actions lead to distinct block structures, indicating localized frequency coupling, while weak actions show sparse and scattered connectivity. In the spatial domain, although both conditions display lower overall connectivity, strong actions still show more defined interaction patterns between channels than weak actions. Meanwhile, across all domains, a pronounced difference (*p*-value < 0.01) in the average clustering coefficient between strong and weak actions can be observed. These findings collectively demonstrate that brain networks derived from multiple domains can effectively represent the brain states of different actions from a multi-domain view.

### 3.5 Hyperparameter studies

This study also explores the impact of various hyperparameters on model performance to optimize the balance between implementation efficiency and computational ACC. Three key hyperparameters—Chebyshev polynomial order, down-sampling rates, and dropouts—are chosen to study. Over the M-GCN, we evaluate DGN by considering Chebyshev polynomial order, down-sampling rate, and dropout from sets [1; 2], [20; 40; 60], and [0.25; 0.5], which are utilized to evaluate M-GCN, respectively. Table 4 presents the scheme of the hyperparameter studies with corresponding decoding results.

**Table 4:** Scheme of the ablation experiment with corresponding decoding results.

**Scheme**	**Order of Chebyshev polynomial**	**Down-sampling factor**	**Dropout**	**ACC**
1-20-0.25	1	20	0.25	69.58%
1-20-0.5	1	20	0.5	70.85%
1-40-0.25	1	40	0.25	71.57%
1-40-0.5	**1**	**40**	**0.5**	**73.60%**
2-20-0.25	2	20	0.25	68.57%
2-20-0. 5	2	20	0.5	69.20%
2-40-0.25	2	40	0.25	70.03%
2-40-0.5	2	40	0.5	72.64%

Bold values indicate the specific scheme that achieved the best performance (highest accuracy) among all the configurations.

The order of the Chebyshev polynomial shows a nuanced effect. Compared with the second order, the first order yields a slight improvement of approximately 1–2%. The down-sampling factor demonstrates a significant impact on model performance. By comparing Scheme 1-40-0.5 and Scheme 1-20-0.5, while keeping all other hyperparameters identical, the ACC increased by 2.75% (*p* = 5.0 × 10^−4^). Meanwhile, the dropout rate exhibits a consistent positive correlation with prediction ACC across different configurations. Under identical conditions, the scheme with a 0.5 dropout achieves a higher ACC than that with a 0.25 dropout. Among all parameter combinations, the configuration with the first-order Chebyshev polynomial, a down-sampling factor of 40, and a dropout rate of 0.5 achieved the best prediction performance. Therefore, Scheme 1-40-0.5 was also adopted in the experiments of this study to ensure optimal performance of M-GCN.

## 4 Discussion

### 4.1 Brain connectivity and brain activation

Brain connectivity and brain activation provide a clear assessment for understanding the neural mechanisms of the cognitive effects of action. Some findings have demonstrated that the strength of connectivity in brain networks reflects functional coordination among task-relevant regions. Si et al. (2021) used the ERP and functional brain network to investigate the neural mechanism underpinning decision-making differences between adults and adolescents. Li et al. (2021) utilized the connectivity of the brain network to study how the brain reconfigures its architecture from the resting state to the stimulus task in the visual oddball task. Distinct connectivity patterns corresponding to different actions are shown in the multi-domain brain network. In both the time-domain and frequency-domain brain networks, the electrodes in the sensorimotor cortex exhibit strong connectivity. It demonstrates that strong actions are associated with enhanced functional interactions between motor-related regions, including the primary motor cortex and premotor areas, as well as coordinated activity with frontal and visual regions.

Analysis of brain activation patterns revealed that different actions elicited distinct activity in specific cortical regions. For instance, strong actions were associated with greater activation in motor-related areas, such as the primary motor cortex and premotor cortex, while frontal and parietal regions were more involved in cognitive processing related to action planning and decision-making. These activation patterns align with the observed connectivity differences, indicating that coordinated network interactions and localized cortical activations jointly contribute to individual differences in MI performance. Building on these findings, future work could explore more complex or diverse action paradigms and further refine brain network analysis methods, such as incorporating additional network features or advanced graph-based models, to capture richer inter-regional interactions. By doing so, it may be possible to better link observed network connectivity patterns with the link between cognitive task and MI, thereby improving both interpretability and predictive performance.

### 4.2 Hyperparameter analysis of M-GCN

The design of hyperparameters plays a crucial role in determining the performance of M-GCN. The first-order Chebyshev polynomial not only achieves slightly higher ACC but also improves computational efficiency by ~30% compared with the second-order setting. This improvement arises because a lower-order approximation effectively captures local neighborhood interactions while avoiding the inclusion of redundant or noisy higher-order information, thus enhancing computational efficiency without compromising discriminative power. The down-sampling factor also demonstrates a significant impact on model performance. According to the Nyquist sampling theorem, the sampling frequency must be at least twice the highest frequency to avoid aliasing. Experimental results demonstrate that a down-sampling factor of 40 effectively preserves ERP components associated with cognitive potentials, thereby enhancing the performance of M-GCN. Regarding dropout, a rate of 0.5 demonstrates an optimal balance between mitigating overfitting and preserving model capacity. This is reflected in an increase in validation ACC compared to lower dropout rates, indicating that moderate regularization is beneficial for stabilizing M-GCN training.

## 5 Conclusion

This study proposes a multi-domain graph convolutional network model for personalized MI action prediction to optimize MI-BCIs performance used for active rehabilitation. The M-GCN extracts multi-domain features to construct multi-domain brain networks using different EEG quantization methods. Moreover, the M-GCN utilizes spectral GCN to learn the topology relationship between EEG channels by analyzing functional connection strength, enabling personalized MI action prediction. By comparing with other models, the proposed M-GCN achieves state-of-the-art performance with 73.60% prediction ACC. This indicates that the M-GCN has adequate prediction performance and model generalization, thereby improving personalized motor imagery prediction. Overall, this study proves that the M-GCN can achieve precise prediction of personalized MI action, reflecting that multi-domain feature fusion based on cognitive task and GCN are effective computational methods for personalized MI action prediction. This research could provide a novel method for personalized BCI.

This study focuses on fusing multi-domain features and decoding cognitive EEG for personalized MI action prediction, while there is space for improvement. However, personalized MI actions exhibit significant differences across multi-domains among different subjects, which may impose challenges for predictive performance across subjects. Meanwhile, the neural basis underlying brain networks derived from cognitive data warrants further investigation. These networks may reflect the coordinated activity among cortical regions, revealing the potential links between cognitive processes and MI. Furthermore, the relatively small number of participants in the current study also limits the generalizability of the findings. The aforementioned issues highlight the necessity of developing new approaches to characterize the effects of actions on the brain. Therefore, strategies such as transfer learning, brain network, and increasing the data size are necessary in future studies.

## Data Availability

The raw data supporting the conclusions of this article will be made available by the authors, without undue reservation.
